# Strength Training vs. Aerobic Training for Managing Pain and Physical Function in Patients with Knee Osteoarthritis: A Systematic Review and Meta-Analysis

**DOI:** 10.3390/healthcare12010033

**Published:** 2023-12-22

**Authors:** Luis Ceballos-Laita, Silvia Lahuerta-Martín, Andoni Carrasco-Uribarren, Sara Cabanillas-Barea, Héctor Hernández-Lázaro, Silvia Pérez-Guillén, Sandra Jiménez-del-Barrio

**Affiliations:** 1 Clinical Research in Health Sciences Group, Department of Surgery, Ophthalmology, Otorhinolaryngology and Physiotherapy, Faculty of Health Sciences, University of Valladolid, 42004 Soria, Spain; luis.ceballos@uva.es (L.C.-L.); silvia.lahuerta@uva.es (S.L.-M.); hector.hernandez.lazaro@uva.es (H.H.-L.); sandra.jimenez.barrio@uva.es (S.J.-d.-B.); 2 Department of Physical Therapy, Faculty of Health Sciences, International University of Catalonia, 08195 Sant Cugat del Vallès, Spain; scabanillas@uic.es (S.C.-B.); sperezgu@uic.es (S.P.-G.)

**Keywords:** knee osteoarthritis, exercise, resistance training, endurance training, systematic review

## Abstract

(1) Background: Strength training (ST) and aerobic training (AT) are the most recommended interventions in patients with knee OA. These recommendations are supported by high-quality evidence, but it is still unknow whether one type of exercise is superior to the other. Thus, the aim was to investigate whether one type of exercise (ST or AT) is superior to the other for improving pain and physical function in patients with knee osteoarthritis. (2) Methods: A systematic review and meta-analysis was carried out following the PRISMA statement. The search strategy was conducted in PubMed, PEDro, Scopus, Web of Science and Cochrane Library databases. Randomized controlled trials comparing ST and AT on pain intensity and physical function in patients with knee osteoarthritis were included. Methodological quality and risk of bias were assessed with a PEDro scale and risk-of-bias tool, respectively. The certainty of evidence was evaluated using GRADE guidelines. (3) Results: Four studies (6 publications) were included. The qualitative and quantitative synthesis showed that ST produces no more improvement in pain intensity (SMD after intervention: 0.02; 95%CI: −0.15, 0.19; I^2^: 0%; three studies; 426 patients) and physical function (SMD after intervention: 0.07; 95%CI: −0.10, 0.24; I^2^: 0%; three studies; 426 patients) compared to AT in patients with knee osteoarthritis. The certainty of evidence was rated as very low. (4) Conclusions: Both type of exercises showed clinical benefits in people with knee osteoarthritis, but no differences between ST and AT were found.

## 1. Introduction

Knee osteoarthritis (OA) is the most important cause of pain and disability in the elderly. Its prevalence has doubled since the mid-20th century [1], and currently the global prevalence has been stated as 16% and its incidence is 203 per 10,000 person—years [2]. Patients with knee OA present a degeneration of all the tissues surrounding the joint, including cartilage, capsule, ligaments, and soft tissues among others. This condition produces inflammation, chronic pain, knee range-of-motion restrictions, muscle weakness, and physical-function limitations [3]. 

Among all the clinical characteristics, muscle weakness seems to be one of the most important risk factors in patients with knee OA. Slemenda et al. and Becker et al. suggested that quadriceps weakness may be a primary risk factor for knee pain, and physical function decrease [4,5]. Muscle weakness seems to contribute directly to the development and progression of the degenerative process in patients with knee OA [4,5], and has shown to be a stronger predictor of disability than X-rays [6]. For these reasons, the most relevant conservative clinical guidelines recommend exercise and physical activity as cornerstones [7,8,9]. 

Despite that, few studies showed that some physical therapists are still using ineffective treatments no longer recommended for the management of knee OA by clinical guidelines; a recent Delphy survey identified that exercise and physical activity interventions were highly prioritized by physical therapists to manage knee OA. Strengthening and aerobic exercise were identified as the most recommended interventions in patients with knee OA. These recommendations are supported by high-quality evidence; however, the physiologic adaptations of each type of exercise are different. Aerobic exercise improves cardiovascular adaptations that increase peak oxygen consumption without significantly changing strength, whereas resistance exercise improves neuromuscular adaptations that increase strength without changing peak oxygen consumption [10]. The different physiological adaptations produced by each type of exercise may generate different effects on patients with knee OA. 

Several well-conducted studies found solid conclusions that strengthening, or aerobic exercise, or its combination decrease pain, improve muscle function, aerobic capacity, physical function, and mood, and reduce risks of comorbidities such as heart disease or diabetes [11,12,13,14,15,16,17,18]. So, clinicians can consider that the most recent evidence shows that both types of exercises are effective to manage the main clinical symptoms of patients with knee OA [19]. But, to the best of our knowledge, only the systematic review developed by Roddy et al. in 2005 compared both types of interventions [20]. This systematic review concluded that both interventions were equally effective for improving pain and physical function in patients with knee OA. However, no quantitative analysis was carried out and new evidence has been published in recent years. Therefore, the aim of this study was to compare whether strength training (ST) is superior to aerobic training (AT) or vice versa for the improvement of pain intensity and physical function in patients with knee OA.

## 2. Materials and Methods

A systematic review was carried out following the Preferred Reporting Items for Systematic Reviews and Meta-Analyses (PRISMA) statement. The study protocol was registered in the International Prospective Register of Systematic Reviews (PROSPERO) with a unique ID CRD42023392468. 

Medline (PubMED), Physiotherapy Evidence Database (PEDro), Cochrane Library, We of Science (WOS), and Scopus databases were searched for potential studies from inception to 21 February 2023. The Population, Intervention, Comparison, Outcome and Study type (PICOS) framework was used to define the search strategy. The following Medical Subject Headings (MeSH) terms were used “knee osteoarthritis”, “resistance training”, “endurance training”, and “pain”. The specific search strategies used in each database are shown in the Appendix A. The snowball search method was used in the reference lists of the potential studies in order to track down related studies that could be finally included. Only studies in English, French, and Spanish were retrieved. 

### 2.1. Eligibility Criteria

The inclusion criteria were based on the PICOS framework: (P) patients diagnosed with knee OA according to an antero-posterior X-ray or to the American College of Rheumatology criteria; (I) interventions based on ST; (C) comparisons based on AT; (O) pain intensity and physical function; (S) randomized controlled trials (RCTs). 

Studies were excluded if (1) the patients were diagnosed with other concomitant conditions that could interfere with the application of active interventions.; (2) the intervention was multimodal or applied a combination of both types of exercise; (3) the outcome of interests were not registered or not valid and reliable instruments were used; (4) they presented no RCT design; (5) they were not published in English, French, Spanish. 

### 2.2. Data Collection Process

After conducting the searches, references from each database were exported to Mendeley desktop and duplicates were removed. Two reviewers (LCL and SJB) assessed the title and abstract of each reference to determine the potential eligibility. Potential full texts were assessed by the same reviewers and a third reviewer (SLM) resolved any discrepancies that might arise during the process.

Data extracted from the studies included information about the study design, sample size, dependent variables, measurement tools, treatment protocol, single session duration, frequency of the intervention, total number of sessions, and main results achieved. A standardized form adapted from the Cochrane Collaboration was used to extract the data. The same two examiners independently extracted the data, and the third examiner solved any discrepancies. 

### 2.3. Methodological Quality and Risk of Bias

Methodological quality of the studies was analyzed using a PEDro scale checklist, and the risk of bias was conducted with the risk-of-bias tool version 2 (RoB2). Although, both instruments assess similar constructs, they cannot be used interchangeably [21]. 

The PEDro scale is based on the Delphi list developed by Verhagen and colleagues [22]. This scale consists of 11 items. A total score out of 10 is derived for each study from the number of criteria that are satisfied. A higher score indicates better methodological quality. A score of 7 or above was considered to be “high” quality, 5–6 was considered “fair” quality and 4 or below was considered “poor quality” [23]. The PEDro scale has shown to be a valid measure of methodological quality of clinical trials and to present an excellent test–retest (r: 0.99) reliability [24]. 

Risk-of-bias version 2 (RoB2) was assessed by the same independent reviewers (LCL and SJB). The RoB 2 tool consist of five questions that assess the following types of bias: risk of bias arising from the randomization process (domine 1), risk of bias due to deviations from the intended interventions (domain 2), risk of bias due to missing outcome data (domain 3), risk of bias in measurement of the outcome (domain 4), and risk of bias in selection of the reported result (domain 5). The responses to signaling questions can be “low”, “unclear”, or “high” for each domain. The official instructions from the Cochrane Collaboration were used to ensure the answer to each question [25]. A study is judged to be at a low risk of bias if all criteria were met, a study was considered as having unclear risk of bias when at least one item presented some concerns, and a study was judged to be of high risk of bias when at least one item was considered as high risk [26].

### 2.4. Certainty of Evidence

The certainty of evidence was assessed using GRADE Evidence Profiles by the same independent reviewers (LC and SJB). The categories of evidence were “high”, “moderate”, “low”, or “very low”, and it helps to have another perspective to researchers and clinicians regarding the importance of the results. The certainty was assessed according to the following domains: risk of bias, inconsistency, indirectness, imprecision, and other considerations.

The certainty of evidence was downgraded in accordance with the presence of the following: risk of bias (downgraded by one level if at least 25% of the participants were from studies with high risk of bias, and two levels if at least 50% of the participants were from studies with high risk of bias: lack of allocation concealment, random allocation and/or sample size calculation, participant, and personnel blinding, blinding of outcome assessors), inconsistency of results (downgraded by one level if the I^2^ value was ≥50%, and two levels if the I^2^ was ≥75%) [27,28], indirectness of evidence (downgraded by one level if different populations, interventions, or comparators were included), and imprecision (downgraded by one level if fewer than 100 participants were included in each group or by two levels If <30 participants were included in each group) [29]. 

### 2.5. Data Synthesis and Analysis

Qualitative and quantitative synthesis was carried out with the following outcome variables: pain and physical function. The qualitative synthesis consisted of the description of the results found in the included studies while the quantitative synthesis consisted of the meta-analysis of the data. 

Two subgroups of meta-analyses were performed for the outcome variables considering the follow-up time points. Outcomes were analyzed based on the post-intervention means and standard deviations (SDs) by calculating the standardized mean difference (SMD) when studies used different scales, with 95% CIs. Significance was set at a *p* value < 0.05. In case these data were not reported in the studies, the authors were contacted by email. The between-group effects size was used to classify the effect estimates as small (SMD at least 0.2 but less than 0.5), medium (SMD from 0.5 to less than 0.8), or large (SMD 0.8 or greater) [30]. 

Data were presented using forest plots. Data were combined in forest plots when at least two trials were considered clinically homogeneous. The studies were considered homogeneous when intervention and outcome variables were similar. When a three-arm study was included, the data from the comparison group were divided [31]. A random-effects meta-analysis was performed when the combination of intervention effects could incorporate an assumption that the studies are not all estimating the same intervention effect [32]. All meta-analyses were conducted using RevMan 5.4. software. 

To detect publication bias and to test each study’s influence, we visually examined the forest plot and performed an exclusion sensitivity analysis. Funnel plots and Begg and Egger tests were not conducted in this study because the meta-analysis did not meet the rule of at least 10 trials included in each forest plot [33].

## 3. Results

**Figure 1:**
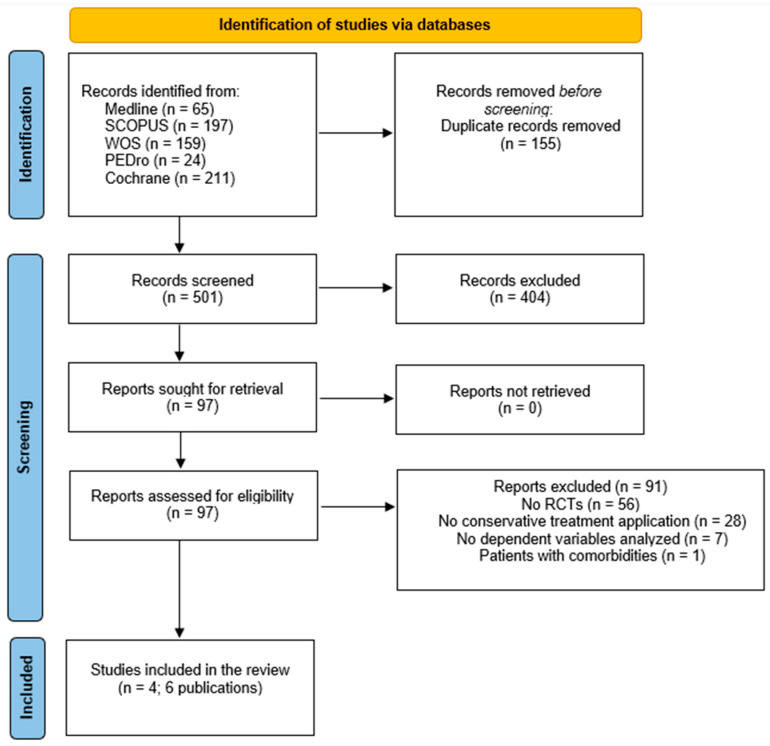
Flowchart diagram.

Initially, a total of 656 studies were obtained from all databases. After removing duplicates, the title and abstract were screened and a total of four studies [34,35,36,37] and two secondary analyses [38,39] (six publications) were included in the systematic review. One study was excluded due to the inclusion of patients with knee OA and other comorbidities [40]. The process of selection is illustrated in the PRISMA flowchart diagram (Figure 1).

### 3.1. Characteristics of the Studies: Sample, Interventions, and Outcomes

The RCTs comprised a total 471 patients with knee OA (patients from secondary analyses were not considered in order to avoid duplication data). The sample size of the studies ranged between 29 and 190 participants. 

All the included studies compared ST versus AT. The ST groups were mainly based on lower limb strengthening applying one to two sets of four to twelve repetitions. The AT groups were mainly based on walking or cycling between 50–85% of the maximum heart rate. The frequency and the total number of sessions varied across all studies. All the studies ranged from three to five session in alternative days per week. The program duration varied across all studies from one month to eighteen months. 

In relation to the assessment of primary outcomes of the studies, all the studies assessed pain using the knee pain score (KPS) (lickert scale), the visual analogue scale (VAS), or the Western Ontario and McMaster Universities Osteoarthritis Index (WOMAC) pain subscale. All the studies assessed physical function using diverse tools such as self-perceived disability, 6 min walking test (6MWT), 30 s chair-to-stand test (30CS), stairs climbing test, WOMAC physical function subscale, or knee injury and osteoarthritis outcomes core (KOOS). All the data about the sociodemographic and clinical characteristics of the studies are shown in the Table 1. 

### 3.2. Methodological Quality and Risk of Bias

According to the PEDro scale, all the studies presented a fair quality with a score of 5 or 6 [34,35,36,38,39], except one study that scored an 8 and was considered as high quality [37]. All the studies failed blinding therapists or patients. It is important to note that therapists cannot be blinded in physical therapy studies because of the nature of the intervention [41]. Some studies presented no allocation concealment, results from less than 85% of the patients initially included, or no statistical comparisons. The methodological quality of each study is shown in the Table 2. 

**Figure 2:**
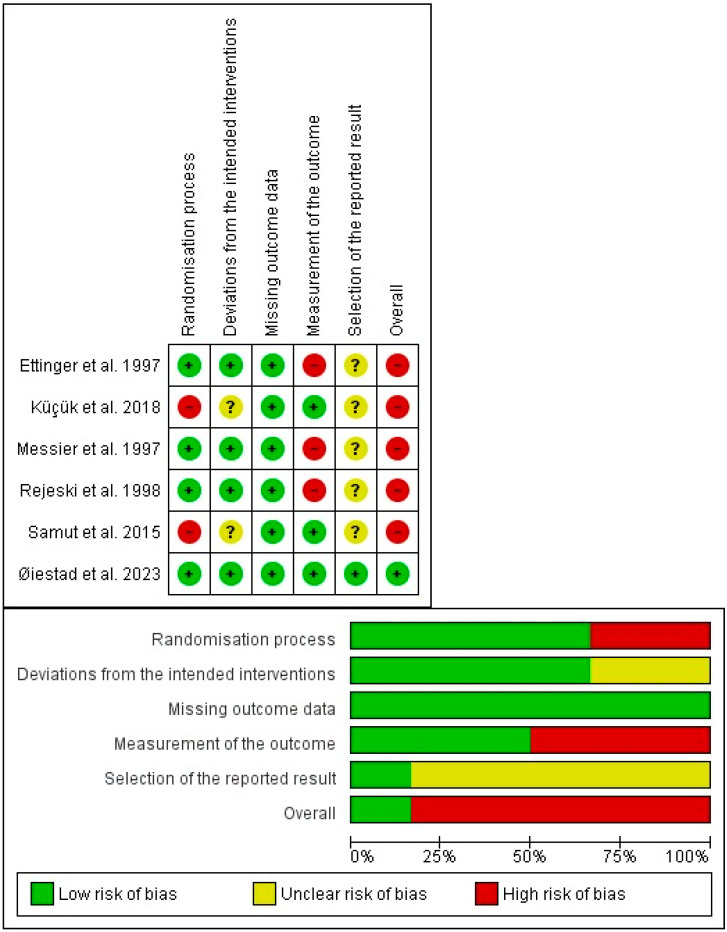
Risk-of-bias tool [34,41,42,43,44,45].

In the risk of bias assessment, all the studies were classified as high risk of bias for not meeting at least one domain [34,35,36,38,39]. Only the study of Øiestad et al. [37] met all the domains and was classified as low risk. The risk of bias of each study is shown in the Figure 2.

**Table 2:** PEDro scale score.

Author	Items											Total Score	
	1	2	3	4	5	6	7	8	9	10	11		
Ettinger et al., 1997 [40]	Y	Y	Y	Y	N	N	Y	N	Y	N	Y	6/10	Fair
Messier et al., 1997 [44]	Y	Y	Y	Y	N	N	Y	N	Y	N	Y	6/10	Fair
Rejeski et al., 1998 [45]	Y	Y	Y	Y	N	N	Y	N	Y	N	N	5/10	Fair
Samut et al., 2015 [41]	Y	Y	N	Y	N	N	N	Y	N	Y	Y	5/10	Fair
Küçük et al., 2018 [42]	Y	Y	N	Y	N	N	N	Y	Y	Y	Y	6/10	Fair
Øiestad et al., 2023 [43]	Y	Y	Y	Y	N	N	Y	Y	Y	Y	Y	8/10	High

Y = criterion satisfied and N = criterion not satisfied. 1, Eligibility criteria was not considered in overall score. 2, Subjects randomly allocated to groups. 3, Allocation was concealed. 4, Groups similar at baseline regarding most important prognostic indicators. 5, Blinding of subjects. 6, Blinding of all therapists. 7, Blinding of all assessors who measured at least one key outcome. 8, Measures of key outcomes obtained from more than 85% of those initially allocated to groups. 9, All subjects for whom outcome measures were available received the treatment or control condition as allocated or where this was not the case, data were analyzed using “intention to treat”. 10, Results of between-group statistical comparisons are reported for at least one key outcome. 11, Study provides both point measures and measures of variability for at least one key outcome.

### 3.3. Effects of Interventions 

Four studies (six publications) were included in the qualitative synthesis, and three were included in the quantitative synthesis. The study of Küçük et al. [42] reported the median, minimum, and maximum values, so it was not possible to include the data in the meta-analysis. 

### 3.4. Pain

**Figure 3:**
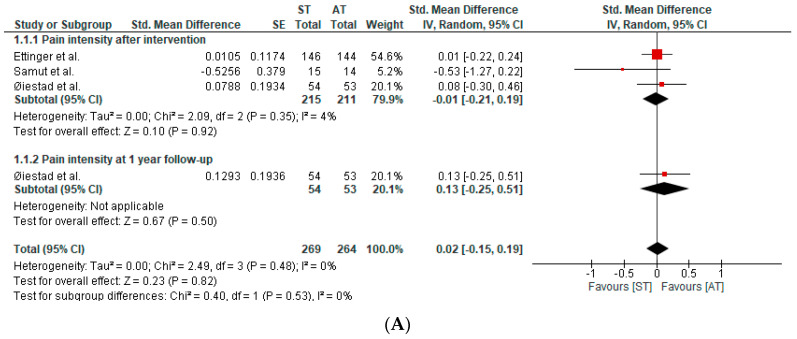

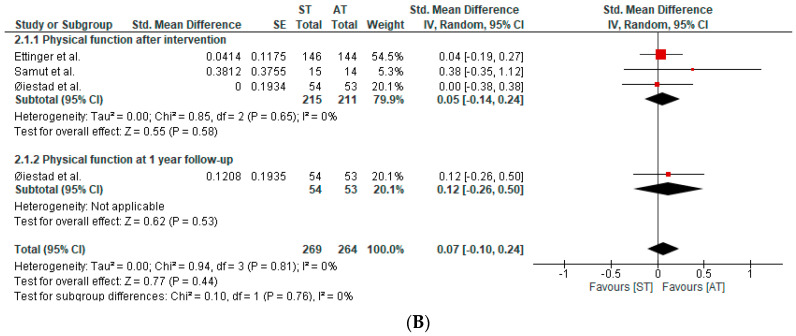
(**A**) Forest plot of pain intensity. (**B**) Forest plot of physical function [40,41,43].

Four studies, in six publications, assessed pain intensity. The qualitative and quantitative synthesis of the results suggested no between-group differences in pain intensity in patients with knee OA (SMD after intervention: −0.01; 95%CI: −0.21, 0.19; I^2^: 4%; three studies; 426 patients; SMD at one-year follow-up: 0.13; 95%CI: −0.25, 0.51; I^2^: 0%; one study; 107 participants) (Figure 3A). The certainty of the evidence was downgraded to very low. 

### 3.5. Physical Function

Four studies, in six publications, assessed physical function. The qualitative and quantitative synthesis of the results suggested no between-group differences in physical function in patients with knee OA (SMD after intervention: 0.05; 95%CI: −0.14, 0.24; I^2^: 0%; three studies; 426 patients; SMD at one-year follow-up: 0.12; 95%CI: −0.26, 0.50; I^2^: 0%; one study; 107 participants) (Figure 3B). The certainty of the evidence was downgraded to very low. 

The overall certainty of evidence was downgraded to very low for pain intensity and physical function. The detailed explanation of the certainty of evidence of each outcome variable is shown in Table 3. 

## 4. Discussion

The aim of this study was to compare whether strength training (ST) is superior to aerobic training (AT) or vice versa for the improvement of pain intensity and physical function in patients with knee OA. The findings of this systematic review and meta-analysis showed no statistically significant differences between ST and AT for managing pain intensity and physical function in patients with knee OA. Both interventions were equally effective for decreasing pain intensity and improving physical function among patients with knee OA. The overall certainty of the evidence was rated as very low. 

In general terms, all the studies showed unanimity achieving no between-group differences in the outcome variables included in this study. Despite this, the results might be influenced by the fair methodological quality and the high risk of bias of most of the included studies. All the studies presented a lack of blinding of patients, therapists, and/or examiners [34,35,36,37,38,39]. Randomized controlled trials involving exercise therapy cannot blind the participants because the intervention is actively carried out by the patient. The lack of therapist blinding is also a common bias in non-pharmacological conservative interventions because therapists cannot be blinded to the treatment they apply [42]. However, stricter randomization processes and concealed allocations, intent-to-treat analyses, and pre-established protocols should be considered in future studies in order to improve the methodological quality. 

In relation to lower limb ST, patients with knee OA have shown not only weakness in the muscles surrounding the knee joint but also in ankle and hip muscles [43]. All the studies applied a lower limb strengthening program, some of them focused on the local muscles and others on the entire lower limb. The within-group analysis or the comparison between the ST and a control group have shown that ST focuses on lower limb muscles which improve pain and physical function in patients with knee OA [34,35,36,38,39]. These results could be because ST increase the cross-sectional area of the muscles surrounding the knee joint [44] and improves lower limb biomechanics [45], which may increase the shock-absorbing capability of a lower extremity during daily living activities [46], reducing the risk of progression of tibiofemoral-joint-space narrowing and cartilage loss [47]. Thus, in summary, ST seems to be effective for decreasing pain and improving physical function in patients with knee OA because muscle strength contributes to load absorption and knee joint stability [48,49].

Concerning AT, recent studies have shown that knee OA is commonly associated with central sensitization [50], which produces higher pain intensity and disability [51]. The central sensitization is defined as “*an increased responsiveness of nociceptive neurons to their normal input, and/or recruitment of a response to normally subthreshold inputs*”. All the studies applied a walking or cycling AT program based on the maximum heart rate [34,35,36,37,38,39]. The within-group analysis or the comparison between the AT and a control group have shown that AT based on walking or cycling exercises improve pain and physical function in patients with knee OA [34,35,36,38,39]. In this sense, AT is the most investigated type of exercise for the management of central sensitization due to the activation of the descending pain-inhibitory mechanism and/or endogenous opioid and cannabinoid systems, and one single session has shown to be effective for reducing pain in patients with chronic pain [52]. Thus, the improvements for pain and physical function achieved in the included studies could be related to the increase in neurotransmitters’ levels, especially, endorphins [53,54,55,56,57].

Regarding the intervention protocols used in each study, it can be concluded that there is a high heterogeneity between them in the training parameters, the session duration, the total number of sessions, and the type of exercises. But, a common recommendation can be made among all the protocol studies. For ST in patients with knee OA, at least one set of eight–twelve repetitions of the local muscles of the knee should be applied during 2 to 3 days per week. For AT in patients with knee OA, cycling or walking at 50–85% of the maximum heart rate should be applied during 2 to 3 days per week. The duration of the programs was too heterogeneous to recommend a minimum dose. 

From a clinical point of view, both active interventions showed a within-group statistically significant improvement or a between group statistically significant improvement compared to a control group. So, ST or AT may be applied in people with knee OA, and improvements in pain intensity and physical function may be expected as long as the clinicians follow the minimum dose recommendations. Probably, future studies could complete the decision making, taking into account other variables in the baseline such as muscle strength, the cross-sectional area of the muscles surrounding the knee joint, and the presence of central sensitization. 

This systematic review has several limitations. First, the heterogeneity of the instrument used to register the outcome variables complicated the interpretation of the results. Second, only patients with knee OA were included, so the results cannot be extrapolated to other osteoarthritic patients. Finally, a selection was not made by the degree of joint degeneration, which could misinterpret the results. 

Future studies should assess the long-term effects of these interventions, the most beneficial intervention time and optimal session duration, as well as the combination of these interventions with other treatments.

## 5. Conclusions

In conclusion, both ST and AT presented clinical benefits for pain intensity and physical function in people with knee OA, which is in accordance with the large body of evidence of other systematic reviews and meta-analysis. But, no between-groups differences were found between both types of exercises in any of the outcomes assessed. The level of evidence was downgraded to very low due to the biases found in the included studies. Future research is needed to establish the proper dose of each type of exercise for patients with knee OA.

## Figures and Tables

**Table 1:** Sociodemographic and clinical variables of the included studies.

Author (Year)	N (Sex Ratio)	Mean Age	ST	AT	Outcome (Tool)	Main Results
Ettinger et al., 1997 [34]	290 (84 M/206 F)	ST: 68 (6) AT: 69 (6)	(n = 146) Protocol: 2 sets/10–12 rep, 9 exercise (upper/lower) 40 m Frequency: 3 d/w Program duration: 18 months (3 month supervised/15 month home-based)	(n = 144) Protocol: 40 min 50-walking 70% HR Frequency: 3 d/w Program duration: 18 months	- KPS (likert) - Self-reported disability - 6MWT - Stair climb	No differences between groups
Messier et al., 1997 (secondary analysis of Ettinger et al.) [39]	67 (17 M/50 F)	ST: 67.2 ± 0.9 AT: 70.3 ± 1.3	(n = 34) Protocol: 2 sets/10–12 rep, 9 exercise (upper/lower) 40 m Frequency: 3 d/w Program duration: 18 months (3 month supervised/15 month home-based)	(n = 33) Protocol: 40 m walking 50–85% HR Frequency: 3 d/w Program duration: 18 months (3 month supervised/15 month home-based)	- KPS (likert)	No differences between groups
Rejeski et al., 1998 (analysis of Ettinger et al.) [38]	357 (104 M/253 F)	NR	Protocol: 2 sets/10–12 rep, 9 exercise (upper/lower) 40 m Frequency: 3 d/w Program duration: 18 months (3 month supervised/15 month home-based)	Protocol: 40 m walking 50–75% HR Frequency: 3 d/w Program duration: 18 months (3 month supervised/15 month home-based)	- KPS (likert) - Stair climb	No differences between groups
Samut et al., 2015 [35]	29 (NR)	ST: 62.46 (7.71) AT: 57.57 (5.79)	(n = 15) Protocol: 5 concentric flexion and extension at angular velocities of 60°, 90°, 120°, 180°/s Frequency: 3 d/w Program duration:1.5 months	(n = 14) Protocol: walking. 1–4 week 65–70% HR/5–6 week 70–75% HR Frequency: 3 d/w Program duration: 1.5 months	- VAS - WOMAC - 30CS - 6MWT	No differences between groups
Küçük et al., 2017 A [36]	45 (45 F)	ST: 51.5 (5) AT: 52.5 (5.3)	(n = 15) Protocol: 10 isokinetic flexion-extension at 60, 90, 120, 150, 180°/s Frequency: 5 d/w Program duration: 1 month	(n = 15) Protocol: 4.5 km/h walking Frequency: 5 d/w Program duration: 1 month	- VAS - WOMAC	No differences between groups
Küçük et al., 2017 B [36]	45 (45 F)	ST: 52.3 (6.9) AT: 52.5 (5.3)	(n = 15) Protocol: 10 reps quadriceps isometric at 90° and 180° Frequency: 5 d/w Program duration: 1 month	(n = 15) Protocol: 4.5 km/h walking Frequency: 5 d/w Program duration: 1 month	- VAS - WOMAC	No differences between groups
Olestad et al., 2023 [37]	107 (49 M/57 F)	ST: 57.6 (6.6) AT: 57.3 (7.1)	(n = 54) Protocol: 1 set/8–12 rep, 6 exercise (lower) Frequency: 2–3 d/w Program duration: 3 months	(n = 53) Protocol: 30 m cycling 70–80% HR Frequency: 2–3 d/w Program duration: 3 months	- VAS - KOOS	No differences between groups

ST: strength training; AT: aerobic training; NR: no reported; HR: heart rate; VAS: visual analog scale; KPS: knee pain score; KOOS: knee injury and osteoarthritis outcomes core; WOMAC: Western Ontario and McMaster Universities Osteoarthritis Index; 6MWT: 6 min walking test; 30CS: 30 s chair to stand test.

**Table 3:** Certainty of evidence according to GRADE recommendations.

Certainty Assesment							No. of Patients		Effect		Certainty	Importance
No. of Studies	Study Design	Risk of Bias	Inconsisntency	Indirectness	Imprecission	Other Considerations	[ST]	[AT]	Relative (95% CI)	Absolute (95% CI)		
Pain intensity												
3	RCTs	Very serious ^a^	not serious	serious ^b^	Not serious	none	215	211	-	SMD 0.02; 95%CI: −0.15, 0.19	⨁◯◯◯ Very low	
Physical function												
3	RCTs	Very serious ^a^	not serious	serious ^b^	Not serious	none	215	211	-	SMD: 0.07; 95%CI: −0.10, 0.24	⨁◯◯◯ Very low	

CI: Confidence interval; SMD: mean difference. Explanations: ^a^ More than 50% of the participants were from studies with poor or fair methodological quality, considering these following aspects: lack of allocation concealment, random allocation and/or sample size calculation, participant and personnel blinding, blinding of outcome assessors. ^b^ The intervention protocols and the duration of the programs were slightly different. High: We are very confident that the true effect is close to the estimate of the effect. Moderate: We are moderately confident in the effect estimate. The true effect is close to the estimate of the effect, but the result can be different. Low: Confidence in the effect estimate is limited; the true effect can be substantially different from the estimate of the effect. Very Low: There is little confidence in the effect estimate; the true effect is likely to be substantially different from the estimate effect.

## Data Availability

Data are contained within the article.

## Appendix A. Search Strategy

### Appendix A.1. Pubmed Search Formula

#1 (“Osteoarthritis, Knee”[Mesh] OR “Knee osteoarthritis”)
#2 (“Resistance Training”[Mesh] OR “Resistance exercise training” OR “Resistance exercise” OR “Strength” OR “Strengthening” OR “Strengthening exercises” OR “Strength training” OR “isometric exercise” OR “quadriceps” OR “gluteus” OR “hamstring” OR “abductors” OR “eccentric” OR “concentric” OR “home-based exercise” OR “home programme” OR “isokinetic”)
#3 (“Endurance Training”[Mesh] OR “aerobic exercise” OR “aerobic exercises” OR “aerobic program” OR “aerobic training” OR “aerobic”)
#4 (“Pain”[Mesh] OR “pain intensity” OR “Function” OR “Physical function” OR “Functional capacity” OR “Physical capacity”)
#5 1# AND 2# AND 3# AND 4#
Results: 65
Data: 29 October 2023

### Appendix A.2. Cochrane Library Search Formula

#1 (Osteoarthritis Knee OR Knee osteoarthritis)
#2 (Resistance Training OR Resistance exercise training OR Resistance exercise OR Strength OR Strengthening OR Strengthening exercises OR Strength training OR isometric exercise OR quadriceps OR gluteus OR hamstring OR abductors OR eccentric OR concentric OR home-based exercise OR home programme OR isokinetic)
#3 (Endurance Training OR aerobic exercise OR aerobic exercises OR aerobic program OR aerobic training OR aerobic)
#4 (Pain OR pain intensity OR Function OR Physical function OR Functional capacity OR Physical capacity)
#5 1# AND 2# AND 3# AND 4#
Results: 211
Data: 29 October 2023

### Appendix A.3. PEDro Search Formula

#1 Knee osteoarthritis AND aerobic
#2 Strength training
#3 lower Leg OR knee
#4 clinical trial
#5 1# AND 2# AND 3# AND 4#
Results: 24
Data: 21 December 2023

### Appendix A.4. Web of Science Search Formula

#1 (“Osteoarthritis Knee” OR “Knee osteoarthritis”)
#2 (“Resistance Training” OR “Resistance exercise training” OR “Resistance exercise” OR “Strength” OR “Strengthening” OR “Strengthening exercises” OR “Strength training” OR “isometric exercise” OR “quadriceps” OR “gluteus” OR “hamstring” OR “adductors” OR “eccentric” OR “concentric” OR “home-based exercise” OR “home programme” OR “isokinetic”)
#3 (“Endurance Training” OR “aerobic exercise” OR “aerobic exercises” OR “aerobic program” OR “aerobic training” OR “aerobic”)
#4 (“Pain” OR “pain intensity” OR “Function” OR “Physical function” OR “Functional capacity” OR “Physical capacity”)
#5 1# AND 2# AND 3# AND 4#
Results: 159
Data: 29 October 2023

### Appendix A.5. Scopus

#1 “Osteoarthritis, Knee” OR “Knee osteoarthritis”
#2 “Resistance Training” OR “Resistance exercise training” OR “Resistance exercise” OR “Strength” OR “Strengthening” OR “Strengthening exercises” OR “Strength training” OR “isometric exercise” OR “quadriceps” OR “gluteus” OR “hamstring” OR “abductors” OR “eccentric” OR “concentric” OR “home-based exercise” OR “home programme” OR “isokinetic”
#3 “Endurance Training” OR “aerobic exercise” OR “aerobic exercises” OR “aerobic program” OR “aerobic training” OR “aerobic”
#4 “Pain” OR “pain intensity” OR “Function” OR “Physical function” OR “Functional capacity” OR “Physical capacity”
#5 1# AND 2# AND 3# AND 4#
Results: 197
Data: 29 October 2023
